# Pet ownership and psychological well-being during the COVID-19 pandemic

**DOI:** 10.1038/s41598-022-10019-z

**Published:** 2022-04-12

**Authors:** Catherine E. Amiot, Christophe Gagné, Brock Bastian

**Affiliations:** 1 Département de Psychologie, Université du Québec À Montréal, C.P. 8888, Succ. Centre-Ville, Montréal, PQ H3C 3P8 Canada; 2 Melbourne School of Psychological Sciences, The University of Melbourne, 06, 05, Redmond Barry Building, Parkville, VIC 3010 Australia

**Keywords:** Psychology, Epidemiology

## Abstract

The question of pet ownership contributing to human well-being has received mixed empirical evidence. This contrasts with the lay intuition that pet ownership contributes positively to wellness. In a large representative sample, we investigate the differences that may exist between pet vs. non-pet owners in terms of their well-being during the COVID-19 pandemic, and examine among different sociodemographic strata, for whom pet ownership can be more vs. less beneficial. A cross-sectional questionnaire survey was conducted among Canadian adults (1220 pet owners, 1204 non-pet owners). Pet owners reported lower well-being than non-pet owners on a majority of well-being indicators; this general pet ownership effect held when accounting for pet species (dogs, cats, other species) and number of pets owned. Compared to owners of other pets, dog owners reported higher well-being. When examining the effect of pet ownership within different socioeconomic strata, being a pet owner was associated with lower well-being among: women; people who have 2 + children living at home; people who are unemployed. Our results offer a counterpoint to popular beliefs emphasising the benefits of pets to human wellness during the COVID-19 pandemic and confirm the importance of accounting for sociodemographic factors to further understand the experience of pet ownership.

## Introduction

The experience of pet ownership exists across many epochs, cultures, and socioeconomic strata^1^. In many occidental countries, such the U.S., Canada, and Australia, a majority (i.e., around 60%) of people live with pets^2–4^. It is commonly assumed that the presence of animals is beneficial to human wellness^5–8^, and this intuition possibly contributed to the increased interest in pet ownership^9^ and demand for adopting a pet observed in Canada during the COVID-19 pandemic^10^. Yet, the scientific literature is divided on the relationship between pet ownership and human health and well-being: Whereas some studies have confirmed the existence of a positive association, others have uncovered null associations or have found a negative relationship (for reviews, see^11–14^). What is needed is rigorous and generalizable evidence to either support or refute these general claims. To this end, the current study drew on a large representative sample of Canadians to investigate the relationship between pet ownership and psychological well-being during the COVID-19 pandemic. We also aimed to provide nuance, by focusing on sociodemographic differences in order to better understand for whom pet ownership may be more or less beneficial at times of high stress, therefore providing an ecologically valid test of the stress buffering effects of pet ownership.

### A contested association between pet ownership and human wellness

Studies conducted across a wide range of scientific disciplines have investigated whether the presence of pets is associated with human health and well-being^11,15^. The general assumption has been that people benefit from the presence of pets^5,16^. Yet, the association between the presence of animals and human wellness is complex and contested. Indeed, some studies have uncovered *positive associations* between the presence of pets and human well-being. For example, in prospective studies, new dog owners reported a significant increase in self-esteem, and children were visited more often by their friends one month following the acquisition of the new pet^17^. In a one-year longitudinal study, older adults who owned a pet maintained participation in more daily activities compared to the non-pet owners^18^. Large epidemiological studies have also revealed that pet owners report fewer physician visits compared to statistically matched non-pet owners^19,20^; see also^21,22^. In experimental studies, hypertensive stockbrokers who were randomly assigned to a pet ownership condition showed, 6 months later, smaller increases in blood pressure during a stressful task compared to non-pet participants^23^. And pet owners whose pet was made physically or cognitively present (i.e., recalled to memory) reported higher aspirations and greater feelings of capability and self-efficacy in attaining personal goals compared to those who were not in the presence of, nor thinking about, their pet^24^.

In contrast, other studies have uncovered *negative links* between the presence of pets and human well-being. For example, in large epidemiological studies, pet owners were found to report lower psychological well-being (e.g., higher anxiety, depression) and perceived health compared to non-pet owners^21,25–27^. Among older Canadian adults, pet owners were less likely than non-owners to report being satisfied with their life^28^. Finally, some studies have *found non-significant associations* between pet ownership and well-being. For instance, in a 6-month prospective study, new pet owners did not report a reduction in their loneliness compared to non-pet owners^29^. Depression levels were also similar for pet and non-pet owners in a nine-year longitudinal study^30^, and in a large population-based study conducted among older adults^31^. Overall, the relationship between pet ownership and wellbeing is far from straightforward.

### Accounting for sociodemographic factors

A growing number of studies are finding that pet owners are not equivalent to non-pet owners in terms of sociodemographic characteristics, with pet owners often from advantaged and majority backgrounds compared to non-pet owners^25,32^. This can potentially lead to an inflation of the positive association, found in some studies, between pet ownership and human wellness^19,33^. In fact, studies that account for sociodemographic factors (e.g., SES, ethnicity, education) have found that such controls attenuate and even erase any positive association between pet ownership and human wellness^33–36^.

To address these methodological issues and provide a systematic and strong test for the role of pets in predicting higher human well-being, the current study recruited a representative sample of Canadian pet owners and non-pet owners; quotas were also used to recruit a relatively equal number of pet and non-pet owners. In addition, poststratification weights are used, which adjust for each subgroup’s (i.e., pet vs. non-pet owners) representativeness relative to the overall Canadian population. Together, these methodological and statistical procedures allow for the improved generalization of findings, and ensure that comparisons of pet vs. non-pet owners are not confounded by sociodemographic differences.

Drawing on data stratified by sociodemographics also allowed us to examine for which subgroups pets are more or less beneficial (see^37^). Indeed, the psychological experience of caring for a pet during a stressful event like a global pandemic may be moderated by social dis/advantage. While on the one hand pet ownership may buffer the stress associated with disadvantage, on the other it may generate added stress, exacerbating vulnerabilities resulting from social inequalities and disadvantage^38^. This could be particularly the case during events such as COVID-19. Indeed, sociodemographic factors have impacted on how people have experienced the COVID-19 pandemic^39^, with some social groups more adversely impacted than others^40–42^.

### The current study

The current study contributes to the current literature in two ways: first, we examine the relationship between pet ownership and well-being drawing on a representative sample, thereby responding to recent calls for more inclusive and diverse research samples when investigating human-pet relations^37,38,43,44^. Second, we draw on sociodemographic factors to determine who is more vs. less likely to benefit from the presence of pets during a highly stressful life experience (i.e., the COVID-19 pandemic). These factors have been identified as theoretically relevant to understanding human-animal relations in general^11^ and as important in research comparing pet and non-pet owners, e.g.,^32,33,38^, and have been found relevant for the experience of the COVID-19 pandemic^40,41^. These factors include: gender, age, area lived in and dwelling type, as well as important indicators of socioeconomic status (i.e., income, employment status) and family size (i.e., number of children living at home). We investigate these associations at a time of high stress, thereby allowing for a critical examination of the role of pets as either buffering stress and isolation or contributing to it by adding an additional source of strain. Given the contradictory prior findings as well as the particularly novel nature of the context in which this study was conducted—i.e., the current COVID-19 pandemic—we adopted an exploratory perspective with respect to the association between pet ownership and psychological well-being; no specific a priori hypothesis is put forward concerning the expected direction of this association.

## Results

### Participants and sociodemographic factors

Data from a total of 2424 participants were analysed, which included 49.5% (1200/2424) males and 50.2% (1218/2424) females (6 indicated ‘other’ for their gender). Whereas 2006 participants (82.8%) completed the questionnaire in English, 418 (17.2%) completed it in French. Approximately 50% of the sample were older than 50 years. More details on the sociodemographic characteristics of the total sample and of the pet and non-pet owner subgroups are presented in Supplementary Table S1.

As can be seen in Table S1, a significantly higher proportion of female respondents were pet owners compared to non-pet owners. Additionally, compared to non-pet owners, a significantly higher proportion of pet owners were found in the 35–39 and 50–54 age groups, but lower proportions were found in the 65 + age groups. A significantly higher proportion of pet owners compared than non pet owners completed the questionnaire in French. Also compared to non-pet owners, a significantly higher proportion of pet owners held a college-level diploma, but lower proportions held a university degree (i.e., Bachelor’s or Master’s). In terms of living arrangements and area, and compared to non-pet owners, higher proportions of pet owners lived in a house or in the countryside, and lower proportions lived in an apartment/condo or in the city. In terms of employment, a higher proportion of pet owners compared to non-pet owners worked full-time. As per ethnicity, a higher proportion of pet owners compared to non-pet owners were Caucasian/White, but lower proportions were of Black/African Canadian or of Asian ethnicities. In terms of marital status, and compared to non-pet owners, a higher proportion of pet owners were in a common-law union, but lower proportions were divorced or single. Many of the differences observed between pet and non-pet owners on these sociodemographic variables align with prior research, e.g.,^25,32^; the higher percentage of pet owners in common-law unions observed in our study could be due to the generally higher prevalence of this type of relationship, which is also considered to be a committed type of relationship, in Canada. When conducting these X^2^ and t-test analyses on the weighted data, these differences became non-significant, confirming that the poststratification weight employed in the current study allowed to ensure that comparisons of pet vs. non-pet owners are not confounded by sociodemographic differences.

### Overall comparison of pet and non-pet owners on the well-being measures

As seen in Table 1, pet owners were found to report lower well-being on 5 of the dependent measures in the ANOVAs. Specifically, pet owners reported lower vitality, lower life satisfaction, and lower presence of life meaning, and higher loneliness compared to non-pet owners. Pet owners were also found to experience higher COVID-related impacts compared to non-pet owners.

**Table 1:** Results of ANOVAs comparing pet and non-pet owners on the psychological well-being and COVID-related impacts measures (weighted data).

	Pet owners		Non-pet owners		Pet ownership effect			
	M	SD	M	SD	dfs	F	η^2^_p_	p
Vitality	4.22	1.33	4.34	1.28	1, 2421	5.34	0.002	0.021
Loneliness	2.29	0.55	2.23	0.52	1, 2418	8.94	0.004	0.003
Life satisfaction	4.35	1.45	4.53	1.38	1, 2418	9.82	0.004	0.002
Presence of life meaning	4.57	1.36	4.75	1.25	1, 2419	11.23	0.005	0.001
Stress	2.79	0.62	2.74	0.57	1, 2416	3.66	0.002	0.056
COVID-related impacts	3.60	1.32	3.44	1.23	1, 2421	9.13	0.004	0.003

Although the current study was designed to specifically compare pet and non-pet owners (i.e., through the use of quotas and the poststratification weight variable), in line with prior research which had investigated the specific effects of cat and dog ownership on human psychological well-being, e.g.,^33,45^, we also investigated whether the pet vs. non-pet owners effects on psychological well-being remain when taking into account the differences between pet species. Specifically, effect coded variables were created to account for the impact of owning these species of pets on well-being. These effect coded variables were then included as covariates in a series of hierarchical multiple regressions (i.e., entered in Block 1) to test if the general pet ownership effects (coded: 0 = non-pet owners; 1 = pet owners; entered in Block 2) reported in Table 1 hold when accounting for the effects of owning dogs, cats, and other types of pets. Specifically, the first effect coded variable (labeled ‘dog ownership’) compared the effect of owning at least one dog (coded as + 1) vs. the effect of owning other types of pets (coded as − 1); non-pet owners in this effect coded variable had a score of zero. The second effect coded variable (labeled ‘cat ownership’) compared the effect of owning at least one cat (coded as + 1) vs. the effect of owning other types of pets (coded as − 1); non-pet owners again had a score of zero. In addition to these effect coded variables, the number of pets participants currently owned was also included as a covariate in Block 1 of the hierarchical multiple regressions. All of the models overall accounted for significant proportions of variance in the well-being measures (*R*^*2*^s ranged from 0.004 to 0.014; *p*s ranged from 0.045 to 0.000).

In these regressions, the general pet ownership effect remained a significant predictor, of: loneliness (*β* = 0.06, *p* = 0.028), life satisfaction (*β* = − 0.08, *p* = 0.001), presence of life meaning (*β* = − 0.09, *p* = 0.000), and COVID-related impacts (*β* = 0.07, *p* = 0.006). While pet ownership was not a significant predictor of vitality (*β* = − 0.03, *p* = 0.328) in these analyses, it become a significant predictor of stress (*β* = 0.06, *p* = 0.036). Addition of the pet ownership variable in Block 2 also resulted in significant increments in *R*^*2*^*s* for the models predicting: loneliness (*R*^*2*^*Δ* = 0.002, *p* = 0.028), life satisfaction (*R*^*2*^*Δ* = 0.004*, p* = 0.001), presence of life meaning (*R*^*2*^*Δ* = 0.005, *p* = 0.000), stress (*R*^*2*^*Δ* = 0.002, *p* = 0.036), and COVID-related impacts (*R*^*2*^*Δ* = 0.003, *p* = 0.006), but not in the model predicting vitality (*R*^*2*^*Δ* = 0.000, *p* = 0.328)*.*

In terms of the covariates, the dog ownership variable was associated significantly with: vitality (*β* = 0.09, *p* = 0.000), loneliness (*β* = − 0.06, *p* = 0.012), life satisfaction (*β* = 0.07, *p* = 0.003), and COVID-related impacts (*β* = − 0.09, *p* = 0.000), but it had no significant effects on: presence of life meaning (*β* = 0.04, *p* = 0.090) and stress (*β* = − 0.001, *p* = 0.959). Hence, compared to owners of other (non dog) pets, dog owners reported higher vitality and life satisfaction, but lower loneliness and COVID-related impacts. The cat ownership variable was not significantly associated with the psychological well-being measures (vitality: *β* = − 0.02, *p* = 0.369; loneliness: *β* = 0.03, *p* = 0.314; life satisfaction: *β* = − 0.03, *p* = 0.322; presence of life meaning: *β* = − 0.01, *p* = 0.734; stress: *β* = 0.05, *p* = 0.051; COVID-related impacts: *β* = − 0.04, *p* = 0.092). Number of pets owned also was not significantly associated with the well-being measures (vitality: *β* = − 0.05, *p* = 0.097; loneliness: *β* = 0.01, *p* = 0.656; life satisfaction: *β* = 0.02, *p* = 0.374; presence of life meaning: *β* = 0.03, *p* = 0.216; stress: *β* = − 0.03, *p* = 0.269; COVID-related impacts: *β* = − 0.00, *p* = 0.951). As a block (Block 1), these covariates accounted for significant proportions of variance in the models predicting: vitality (*R*^*2*^*Δ* = 0.014, *p* = 0.000), loneliness (*R*^*2*^*Δ* = 0.008, *p* = 0.000), life satisfaction (*R*^*2*^*Δ* = 0.008, *p* = 0.000), and COVID-related impacts (*R*^*2*^*Δ* = 0.006, *p* = 0.002), but not in the models predicting: presence of life meaning (*R*^*2*^*Δ* = 0.002, *p* = 0.137) and stress (*R*^*2*^*Δ* = 0.002, *p* = 0.147).

### Comparing pet and non-pet owners at different levels of the sociodemographic variables

The next series of ANOVAs, presented in Tables 2, 3, 4, 5, 6, 7 and 8, explored the possible differences between pet and non-pet owners at the different levels of the sociodemographic variables. For these analyses, we focused specifically on the comparison of pet and non-pet owners, allowing us to use the poststratification weight variable. The analyses revealed a number of moderators of the main effects presented above.

**Table 2:** Results of ANOVAs comparing pet and non-pet owners across genders on the well-being and COVID-related impacts variables (weighted data).

	Pet owners				Non-pet owners				Pet ownership		Gender		Pet ownership × gender	
	Men		Women		Men		Women							
	M	SD	M	SD	M	SD	M	SD	F	η^2^_p_	F	η^2^_p_	F	η^2^_p_
Vitality	4.47	1.35	4.00^a^	1.26	4.44	1.12	4.26^a^	1.43	4.54*	0.002	39.04***	0.016	7.37**	0.003
Loneliness	2.27	0.54	2.31^a^	0.55	2.26	0.48	2.19^a^	0.56	8.25**	0.003	0.56	0.000	6.54*	0.003
Life satisfaction	4.51	1.43	4.23^a^	1.45	4.40	1.28	4.68^a^	1.47	8.59**	0.004	0.00	0.000	23.67***	0.010
Presence of life meaning	4.60	1.37	4.56^a^	1.34	4.66	1.15	4.84^a^	1.35	10.56**	0.004	1.79	0.001	4.63*	0.002
Stress	2.70	0.59	2.86^a^	0.62	2.70	0.52	2.78^a^	0.62	3.07	0.001	25.77***	0.011	3.08	0.001
COVID-related impacts	3.38	1.35	3.78^a^	1.26	3.35	1.18	3.51^a^	1.27	8.19**	0.003	28.59***	0.012	5.13*	0.002

*p < 0.05; **p < 0.01; ***p < 0.001. Participants (*n* = 6) who indicated ‘other’ as their gender were not included in these analyses given this cell size. Within a row, means with the same superscript differ in the paired comparison analyses comparing pet and non-pet owners at each level of the demographic variable (*p* < 0.05). Only when the interaction is significant are these paired comparisons interpreted.

**Table 3:** Results of ANOVAs comparing pet and non-pet owners across age categories on the well-being and COVID-related impacts variables (weighted data).

	Pet owners						Non-pet owners						Pet ownership			Age			Pet ownership × age	
	Young adults		Adults		Seniors		Young adults		Adults		Seniors									
	M	SD	M	SD	M	SD	M	SD	M	SD	M	SD	F	η^2^_p_	F		η^2^_p_	F		η^2^_p_
Vitality	3.91	1.18	4.18^a^	1.27	4.51	1.69	4.07	1.40	4.32^a^	1.28	4.55	1.21	2.81	0.001	16.52***		0.013	0.39		0.000
Loneliness	2.40	0.45	2.32^a^	0.52	2.15	0.72	2.43	0.39	2.24^a^	0.52	2.09	0.51	1.93	0.001	31.26***		0.025	1.22		0.001
Life satisfaction	4.25	1.33	4.27^a^	1.35	4.68	2.00	4.15	1.32	4.54^a^	1.40	4.72	1.34	0.86	0.000	13.24***		0.011	2.76		0.002
Presence of life meaning	4.28	1.29	4.46^a^	1.25	5.05	1.82	4.27	1.35	4.72^a^	1.25	5.07	1.14	1.77	0.001	38.73***		0.031	2.39		0.002
Stress	2.99	0.49	2.84^a^	0.57	2.52	0.84	3.10	0.44	2.77^a^	0.56	2.47	0.55	0.02	0.000	94.87***		0.073	2.81		0.002
COVID-related impacts	3.71^a^	1.11	3.66^b^	1.29	3.33^c^	1.59	4.09^a^	1.34	3.46^b^	1.23	3.03^c^	1.02	0.32	0.000	31.91***		0.026	7.12***		0.006

*p < 0.05; **p < 0.01; ***p < 0.001. The age categories used were based on Statistics Canada: young adults: 18–24 (*n* = 251), adults: 25–64 (*n* = 1740), seniors: 65 + (*n* = 433). (https://www.statcan.gc.ca/eng/concepts/definitions/age2). Within a row, means with the same superscript differ in the paired comparison analyses comparing pet and non-pet owners at each level of the demographic variable (*p* < 0.05). Only when the interaction is significant are these paired comparisons interpreted.

**Table 4:** Results of ANOVAs comparing pet and non-pet owners across areas lived in on the well-being and COVID-related impacts variables (weighted data).

	Pet owners						Non-pet owners						Pet ownership		Area		Pet ownership × area	
	City		Suburb		Countryside		City		Suburb		Countryside							
	M	SD	M	SD	M	SD	M	SD	M	SD	M	SD	F	η^2^_p_	F	η^2^_p_	F	η^2^_p_
Vitality	4.15 ^a^	1.39	4.32	1.27	4.20	1.27	4.36 ^a^	1.19	4.28	1.27	4.42	1.75	4.84*	0.002	0.43	0.000	2.59	0.002
Loneliness	2.34^a^	0.55	2.24	0.55	2.24	0.52	2.24^a^	0.46	2.21	0.52	2.22	0.73	4.46*	0.002	4.30*	0.004	1.50	0.001
Life satisfaction	4.17^a^	1.49	4.51	1.45	4.58	1.29	4.39^a^	1.25	4.61	1.44	4.82	1.77	8.33**	0.003	16.83***	0.014	0.63	0.001
Presence of life meaning	4.49^a^	1.37	4.71	1.39	4.51^b^	1.24	4.67^a^	1.19	4.71	1.25	5.05^b^	1.54	17.31***	0.007	4.39*	0.004	5.61**	0.005
Stress	2.86^a^	0.61	2.73	0.62	2.68	0.61	2.79^a^	0.49	2.71	0.59	2.68	0.83	1.71	0.001	12.40***	0.010	0.73	0.001
COVID-related impacts	3.73 ^a^	1.37	3.53	1.28	3.32	1.22	3.55 ^a^	1.22	3.44	1.16	3.11	1.40	7.93**	0.003	16.44***	0.013	0.42	0.000

*p < 0.05; **p < 0.01; ***p < 0.001. City: *n* = 1236, Suburb: *n* = 848, Countryside: *n* = 340. Within a row, means with the same superscript differ in the paired comparison analyses comparing pet and non-pet owners at each level of the demographic variable (*p* < 0.05). Only when the interaction is significant are these paired comparisons interpreted.

**Table 5:** Results of ANOVAs comparing pet and non-pet owners across dwelling types on the well-being and COVID-related impacts variables (weighted data).

	Pet owners				Non-pet owners				Pet ownership		Dwelling type		Pet ownership × dwelling type	
	Apartment/condo		House		Apartment/condo		House							
	M	SD	M	SD	M	SD	M	SD	F	η^2^_p_	F	η^2^_p_	F	η^2^_p_
Vitality	3.92^a^	1.44	4.33	1.27	4.34^a^	1.15	4.34	1.36	13.40***	0.006	11.93***	0.005	11.61***	0.005
Loneliness	2.40^a^	0.59	2.25	0.53	2.28^a^	0.43	2.21	0.56	11.65***	0.005	18.79***	0.008	2.65	0.001
Life satisfaction	3.88^a^	1.68	4.52	1.34	4.23^a^	1.18	4.64	1.48	13.28***	0.005	65.31***	0.026	3.20	0.001
Presence of life meaning	4.30^a^	1.49	4.66^b^	1.30	4.58^a^	1.11	4.81^b^	1.33	12.08***	0.005	23.91***	0.010	1.17	0.000
Stress	2.85	0.66	2.77	0.60	2.81	0.44	2.72	0.64	2.55	0.001	8.90**	0.004	0.03	0.000
COVID-related impacts	3.83 ^a^	1.46	3.51 ^b^	1.26	3.59 ^a^	1.07	3.39 ^b^	1.32	9.81**	0.004	19.20***	0.008	0.98	0.000

*p < 0.05; **p < 0.01; ***p < 0.001. The House category (*n* = 1663) included participants who were living in a house or other types of dwelling (e.g., townhouse, duplex, mobile home); Apartment/Condo (*n* = 761). These two dwelling categories were also used in the poststratification weight variable. Within a row, means with the same superscript differ in the paired comparison analyses comparing pet and non-pet owners at each level of the demographic variable (*p* < 0.05). Only when the interaction is significant are these paired comparisons interpreted.

**Table 6:** Results of ANOVAs comparing pet and non-pet owners across income levels on the well-being and COVID-related impacts variables (weighted data).

	Pet owners						Non-pet owners						Pet ownership		Income		Pet ownership × income	
	0–99 K		100–199 K		200 K +		0–99 K		100–199 K		200 K +							
	M	SD	M	SD	M	SD	M	SD	M	SD	M	SD	F	η^2^_p_	F	η^2^_p_	F	η^2^_p_
Vitality	4.17	1.39	4.39	1.10	4.59	1.54	4.28	1.33	4.55	1.11	4.52	1.66	0.81	0.000	10.48***	0.009	0.54	0.000
Loneliness	2.33	0.56	2.23^a^	0.44	2.04	0.72	2.31	0.51	2.06^a^	0.43	2.02	0.65	4.96*	0.002	38.57***	0.034	3.84*	0.003
Life satisfaction	4.23	1.52	4.62^a^	1.91	5.11	1.62	4.34	1.39	4.96^a^	1.19	5.18	1.53	4.18*	0.002	48.47***	0.042	1.43	0.001
Presence of life meaning	4.52	1.40	4.74^a^	1.13	4.88	1.83	4.62	1.30	4.98^a^	1.05	5.21	1.48	8.11**	0.004	17.25***	0.015	0.96	0.001
Stress	2.82	0.66	2.72^a^	0.50	2.67	0.75	2.80	0.56	2.60^a^	0.56	2.65	0.76	2.27	0.001	15.87***	0.014	1.53	0.001
COVID-related impacts	3.75^a^	1.40	3.39	1.09	3.35	1.25	3.45^a^	1.31	3.24	1.02	3.70	1.38	0.15	0.000	10.10***	0.009	5.41**	0.005

*p < 0.05; **p < 0.01; ***p < 0.001. These gross annual household income categories were also used in the poststratification weight variable: 0-99 K: *n* = 1510, 99 K-199 K: *n* = 614, 200 K + : *n* = 89. Within a row, means with the same superscript differ in the paired comparison analyses comparing pet and non-pet owners at each level of the demographic variable (*p* < 0.05). Only when the interaction is significant are these paired comparisons interpreted.

**Table 7:** Results of ANOVAs comparing pet and non-pet owners across employment status on the well-being and COVID-related impacts variables (weighted data).

	Pet owners								Non-pet owners								Pet ownership		Employment status		Pet ownership × employment status	
	Full-time		Part-time		Unemployed		Other		Full-time		Part-time		Unemployed		Other							
	M	SD	M	SD	M	SD	M	SD	M	SD	M	SD	M	SD	M	SD	F	η^2^_p_	F	η^2^_p_	F	η^2^_p_
Vitality	4.33	1.24	4.10	1.19	3.37	1.16	4.25^a^	1.50	4.43	1.22	4.02	1.32	3.56	1.48	4.46^a^	1.24	2.03	0.001	24.74***	0.030	0.90	0.001
Loneliness	2.27	0.51	2.34	0.52	2.69^a^	0.49	2.24^b^	0.60	2.23	0.48	2.32	0.56	2.47^a^	0.53	2.14^b^	0.52	9.68**	0.004	24.31***	0.029	1.95	0.002
Life satisfaction	4.56	1.27	4.08	1.29	2.70^a^	1.28	4.43^b^	1.63	4.59	1.32	4.24	1.51	3.45^a^	1.46	4.73^b^	1.31	15.99***	0.007	57.63***	0.067	3.75*	0.005
Presence of life meaning	4.68	1.23	4.46	1.16	3.38^a^	1.30	4.65^b^	1.55	4.67	1.19	4.69	1.35	4.04^a^	1.37	4.98^b^	1.22	17.83***	0.007	30.62***	0.037	4.74**	0.006
Stress	2.79	0.53	2.87	0.52	3.32^a^	0.70	2.67^b^	0.70	2.78	0.52	2.89	0.61	3.08^a^	0.55	2.58^b^	0.58	5.76*	0.002	46.94***	0.055	2.31	0.003
COVID-related impacts	3.63 ^a^	1.31	3.75	1.15	4.35	1.33	3.38	1.34	3.47 ^a^	1.27	3.50	1.14	4.09	1.28	3.27	1.16	7.47**	0.003	22.53***	0.027	0.31	0.000

*p < 0.05; **p < 0.01; ***p < 0.001. Participants employed full-time: *n* = 1157; employed part-time: *n* = 305; unemployed: *n* = 180; and other statuses (i.e., students, homemakers, retired): *n* = 782. These employment status categories were also used in the poststratification weight variable. Within a row, means with the same superscript differ in the paired comparison analyses comparing pet and non-pet owners at each level of the demographic variable (*p* < 0.05). Only when the interaction is significant are these paired comparisons interpreted.

**Table 8:** Results of ANOVAs comparing pet and non-pet owners across number of children living at home on the well-being and COVID-related impacts variables (weighted data).

	Pet owners								Non-pet owners								Pet ownership		Number of children		Pet ownership × number of children	
	0 Children		1 Child		2 Children		3 + Children		0 Children		1 Child		2 Children		3 + Children							
	M	SD	M	SD	M	SD	M	SD	M	SD	M	SD	M	SD	M	SD	F	η^2^_p_	F	η^2^_p_	F	η^2^_p_
Vitality	4.13	1.37	4.46	1.15	4.14^a^	1.32	4.47	1.41	4.22	1.19	4.53	1.50	4.44^a^	1.60	4.69	1.18	5.65*	0.002	10.25***	0.013	0.88	0.001
Loneliness	2.31	0.56	2.20	0.50	2.27	0.55	2.39^a^	0.56	2.27	0.47	2.10	0.62	2.27	0.61	2.04^a^	0.53	17.73***	0.007	8.89***	0.011	4.65**	0.006
Life satisfaction	4.20	1.47	4.70	1.31	4.47	1.34	4.45^a^	1.79	4.33	1.32	4.81	1.45	4.69	1.57	5.19^a^	1.39	15.59***	0.006	20.16***	0.024	2.40	0.003
Presence of life meaning	4.45	1.41	4.77^a^	1.23	4.68^b^	1.17	4.79^c^	1.66	4.53	1.16	5.02^a^	1.42	4.99^b^	1.39	5.29^c^	1.40	16.77***	0.007	19.83***	0.024	1.94	0.002
Stress	2.76	0.64	2.72	0.54	2.92^a^	0.53	2.96^b^	0.78	2.76	0.52	2.66	0.71	2.78^a^	0.70	2.73^b^	0.73	10.66**	0.004	6.16***	0.008	2.78*	0.003
COVID-related impacts	3.51	1.26	3.60	1.23	3.83	1.51	3.79^a^	1.72	3.43	1.13	3.39	1.45	3.63	1.53	3.19^a^	1.47	15.98***	0.007	4.35**	0.005	2.31	0.003

*p < 0.05; **p < 0.01; ***p < 0.001. Zero children living at home: *n* = 1638; 1 child living at home: *n* = 417; 2 children living at home: *n* = 268; 3 or more children living at home: *n* = 101. These categories were also used in the poststratification weight variable. Within a row, means with the same superscript differ in the paired comparison analyses comparing pet and non-pet owners at each level of the demographic variable (*p* < 0.05). Only when the interaction is significant are these paired comparisons interpreted.

#### Gender

As seen in Table 2, gender had significant main effects on vitality, stress, and COVID-related impacts: women reported lower vitality but higher stress and COVID-related impacts compared to men. These results align with those of prior epidemiological studies conducted among the Canadian population^46,47^. Five significant interactions also emerged. In terms of vitality, whereas male pet owners and male non-pet owners did not differ in their levels of vitality (*F*(1, 2413) = 0.17, *p* = 0.684, *η*^*2*^_*p*_ = 0.000), female pet owners reported lower vitality compared to female non-pet owners (*F*(1, 2413) = 12.14), *p* < 0.001, *η*^*2*^_*p*_ = 0.005). The same pattern was observable on the loneliness measure, with male pet owners and male non-pet owners showing no difference in loneliness (*F*(1, 2410) = 0.05, *p* = 0.827, *η*^*2*^_*p*_ = 0.000), whereas female pet owners reported higher loneliness compared to female non-pet owners (*F*(1, 2410) = 15.25, *p* < 0.001, *η*^*2*^_*p*_ = 0.006). On the life satisfaction measure, male pet owners and male non-pet owners showed no difference (*F*(1, 2410) = 1.81, *p* = 0.179, *η*^*2*^_*p*_ = 0.001), whereas female pet owners reported lower life satisfaction compared to female non-pet owners (*F*(1, 2410) = 31.44, *p* < 0.001, *η*^*2*^_*p*_ = 0.013). For the presence of life meaning, again male pet owners and male non-pet owners showed no difference (*F*(1, 2411) = 0.58, *p* = 0.445, *η*^*2*^_*p*_ = 0.000), whereas female pet owners reported lower presence of life meaning compared to female non-pet owners (*F*(1, 2411) = 15.09, *p* < 0.001, *η*^*2*^_*p*_ = 0.006). Finally, female pet owners reported experiencing higher COVID-related impacts than female non-pet owners (*F*(1, 2413) = 13.60, *p* < 0.001, *η*^*2*^_*p*_ = 0.006), whereas males did not differ, whether they were pet owners or non-pet owners, in terms of COVID-related impacts (*F*(1, 2413) = 0.17, *p* = 0.678, *η*^*2*^_*p*_ = 0.000). We return to a conceptual discussion of these results, which may be due to the increased strain associated with pet ownership experienced by certain strata of the Canadian population during the COVID-19 pandemic, in the Discussion section below.

#### Age

Main effects of age emerged on all of the dependent variables (see Table 3), globally showing higher well-being among seniors (65 +), followed by adults (25–64), and then by young adults (18–24), in the paired comparisons (all *p*s ≤ 0.029). This pattern emerged on: vitality (*M*s = 4.53, 4.25, 3.99; *SD*s = 1.39, 1.27, 1.29, respectively); loneliness (*M*s = 2.12, 2.28, 2.42; *SD*s = 0.59, 0.52, 0.42, respectively); life satisfaction (*M*s = 4.70 4.40, 4.20; *SD*s = 1.59, 1.38, 1.32, respectively); presence of life meaning (*M*s = 5.06, 4.59, 4.28; *SD*s = 1.41, 1.26, 1.31, respectively); stress (*M*s = 2.49, 2.81, 3.05; *SD*s = 0.66, 0.56, 0.47, respectively); and COVID-related impacts (*M*s = 3.18, 3.56, 3.90; *SD*s = 1.25, 1.27, 1.24, respectively). These age differences align with prior research^48^. One interaction emerged on the COVID-related impacts variable, revealing that adult pet owners reported higher COVID-related impacts compared to adult non-pet owners (*F*(1, 2417) = 10.35, *p* = 0.001, *η*^*2*^_*p*_ = 0.004). Similarly, senior pet owners reported higher COVID-related impacts compared to senior non-pet owners (*F*(1, 2417) = 7.16, *p* = 0.008, *η*^*2*^_*p*_ = 0.003), although this difference was attenuated in this age group. In contrast, among young adults, pet owners were found to report lower COVID-related impacts compared to non-pet owners (*F*(1, 2417) = 6.13, *p* = 0.013, *η*^*2*^_*p*_ = 0.003), suggesting that owning a pet may buffer against the stress generated by the COVID context for younger people.

#### Area lived in

Area lived in was associated with five of the dependent variables (see Table 4), globally showing in the paired comparisons (all relevant *p*s ≤ 0.043), that people living in the countryside and in the suburbs reported higher well-being compared to people living in the city. This pattern emerged on loneliness (*M*s = 2.23, 2.23, 2.29; *SD*s = 0.60, 0.54, 0.51, respectively); life satisfaction (*M*s = 4.70 4.56, 4.28; *SD*s = 1.48, 1.45, 1.37, respectively); presence of life meaning (*M*s = 4.78, 4.71, 4.58; *SD*s = 1.38, 1.33, 1.28, respectively); and stress (*M*s = 2.68, 2.72 2.83; *SD*s = 0.70, 0.61, 0.55, respectively). These main effects observed for area align with findings showing that being surrounded by nature, which is generally more accessible when living in the countryside and the suburbs, is associated with higher well-being^49^. For the COVID-related impacts variable, people in the city reported experiencing higher levels of stress (*M* = 3.64; *SD* = 1.30), followed by people living in the suburbs (*M* = 3.48; *SD* = 1.23), and by people living in the countryside (*M* = 3.21; *SD* = 1.29). Only one significant interaction emerged, on presence of life meaning, showing that non-pet owners living in the city and in the countryside reported higher presence of life meaning compared to pet owners living in the city (*F*(1, 2415) = 6.17, *p* = 0.013, *η*^*2*^_*p*_ = 0.003) and in the countryside (*F*(1, 2415) = 16.32, *p* < 0.001, *η*^*2*^_*p*_ = 0.007).

#### Dwelling type

As seen in Table 5, dwelling type also had significant main effects on all dependent measures: Participants living in a house reported higher vitality (*M* = 4.33; *SD* = 1.31), life satisfaction (*M* = 4.58; *SD* = 1.40), and presence of life meaning (*M* = 4.73; *SD* = 1.32), but lower loneliness (*M* = 2.23; *SD* = 0.54), stress (*M* = 2.74; *SD* = 0.62), and COVID-related impacts (*M* = 3.45; *SD* = 1.29) compared to people living in an apartment or condo (*M*s = 4.13, 4.06, 4.44, 2.34, 4.52, 2.83, 3.49, 3.71; *SD*s = 1.28, 1.40, 1.28, 0.50, 1.26, 0.54, 1.12, 1.24, respectively). These dwelling type main effects align with prior research^50^. Only one significant interaction emerged on vitality, showing that pet owners living in an apartment or condo reported lower vitality compared to non-pet owners living in an apartment or condo (*F*(1, 2419) = 16.94, *p* < 0.001, *η*^*2*^_*p*_ = 0.007); participants who live in a house did not differ whether they are pet owners or non-pet owners in their levels of vitality (*F*(1, 2419) = 0.06, *p* = 0.804, *η*^*2*^_*p*_ = 0.000). This suggests a generally modest role for dwelling type in moderating the experience of pet ownership.

#### Gross yearly family income

Main effects of income emerged on all dependent variables (see Table 6), globally showing, in the paired comparisons, higher well-being as the income category increased, with the most consistent significant increase observed between the 0-99 K and the 100 K-199 K categories (all relevant *p*s < 0.001 in the paired comparisons). This pattern emerged on: vitality (*M*s = 4.22, 4.47, 4.56; *SD*s = 1.36, 1.11, 1.58, respectively for 0-99 K, 100 K-199 K, and 200 K + , with a significant difference between the 0-99 K and the 100 K-199 K categories: *p* < 0.001); loneliness (*M*s = 2.32, 2.15, 2.03; *SD*s = 0.54, 0.44, 0.69, respectively, with significant differences between the 0-99 K and the 100 K-199 K categories: *p* < 0.001, and the 100 K-199 K and 200 K + categories: *p* = 0.013); life satisfaction (*M*s = 4.28 4.79, 5.15; *SD*s = 1.45, 1.20, 1.57, respectively, with significant differences between the 0-99 K and the 100 K-199 K categories: *p* < 0.001, and the 100 K-199 K and 200 K + categories: *p* = 0.003); presence of life meaning (*M*s = 4.57, 4.86, 5.04; *SD*s = 1.35, 1.10, 1.69, respectively, with a significant difference between the 0-99 K and the 100 K-199 K categories: *p* < 0.001); stress (*M*s = 2.81, 2.66, 2.66; *SD*s = 0.61, 0.53, 0.75, respectively, with a significant difference between the 0-99 K and the 100 K-199 K categories: *p* < 0.001); and COVID-related impacts (*M*s = 3.60, 3.31, 3.52; *SD*s = 1.36, 1.06, 1.33, respectively, with a significant difference between the 0-99 K and the 100 K-199 K categories: *p* < 0.001). These income effects align with prior research showing a positive association between SES (including income) and well-being^51,52^. Two interactions reached significance. In terms of loneliness, pet owners in the 100-199 K income category reported higher loneliness than non-pet owners in this income category (*F*(1, 2203) = 13.83, *p* < 0.001, *η*^*2*^_*p*_ = 0.006); pet and non-pet owners did not differ in their levels of loneliness in the two other income categories (0-99 K: *F*(1, 2203) = 0.80, *p* = 0.372, *η*^*2*^_*p*_ = 0.000; 200 K + : *F*(1, 2203) = 0.06, *p* = 0.802, *η*^*2*^_*p*_ = 0.000. The interaction observed on the COVID-related impacts variable revealed that pet owners in the 0-99 K income category reported experiencing higher COVID-related impacts compared to non-pet owners in this income category (*F*(1, 2207) = 20.51, *p* < 0.001, *η*^*2*^_*p*_ = 0.009); pet and non-pet owners did not differ in their levels of experiencing COVID-related impacts in the other two income categories (100 K-199 K: *F*(1, 2207) = 1.80, *p* = 0.180, *η*^*2*^_*p*_ = 0.001; 200 K + : *F*(1, 2207) = 3.44, *p* = 0.064, *η*^*2*^_*p*_ = 0.002). This last finding also points to a possible strain effect associated with pet ownership and experienced more by certain segments of the Canadian population during the COVID-19 pandemic; it will be further discussed in the Discussion section below.

#### Employment status

Main effects for employment type emerged on all dependent variables (see Table 7), globally showing, in the paired comparisons, that being unemployed is associated with lower well-being compared to the 3 other employment status categories (all *p*s < 0.001 for these specific comparisons). These employment status main effects align with (yet could also be confounded with) the income main effects reported above. Two significant interactions also emerged. The significant interaction observed on life satisfaction shows that pet owners who are unemployed reported lower life satisfaction compared to non-pet owners who are unemployed (*F*(1, 2412) = 10.65, *p* = 0.001, *η*^*2*^_*p*_ = 0.004). Furthermore, pet owners who have other employment statuses (i.e., who are students, homemakers, retired) reported lower life satisfaction compared to non-pet owners who have these other employment statuses (*F*(1, 2412) = 10.06, *p* = 0.002, *η*^*2*^_*p*_ = 0.004). Pet and non-pet owners who work full time or part time did not differ in terms of their life satisfaction (*F*(1, 2412) = 0.14, *p* = 0.711, *η*^*2*^_*p*_ = 0.000; *F*(1, 2412) = 0.92, *p* = 0.338, *η*^*2*^_*p*_ = 0.000, respectively). The significant interaction observed on presence of life meaning also reveals that pet owners who are unemployed reported lower meaning in life compared to non-pet owners who are unemployed (*F*(1, 2413) = 9.41, *p* = 0.002, *η*^*2*^_*p*_ = 0.004). As well, pet owners who have other employment statuses reported lower presence of life meaning compared to non-pet owners who have these other employment statuses (*F*(1, 2413) = 14.34, *p* < 0.001, *η*^*2*^_*p*_ = 0.006). Pet and non-pet owners who work full time or part time did not differ in terms of presence of life meaning (*F*(1, 2413) = 0.01, *p* = 0.943, *η*^*2*^_*p*_ = 0.000; *F*(1, 2413) = 2.28, *p* = 0.131, *η*^*2*^_*p*_ = 0.001, respectively). These findings also point to a possible strain effect, and suggest that pet ownership could represent more of a burden among certain strata of the Canadian population (i.e., people who are unemployed and those in more instable situations). We come back to a discussion of these findings in the Discussion section.

#### Number of children living at home

Main effects of number of children living at home emerged on all of the dependent variables (see Table 8). Globally, the trend whereby having at least one child living at home is associated with higher well-being was observed specifically on the life satisfaction and presence of life meaning measures (all *p*s < 0.001 for these specific comparisons). Two significant interactions also emerged. The significant interaction observed on loneliness shows that pet owners who have 3 or more children living with them at home reported higher loneliness compared to non-pet owners who have 3 or more children living with them at home (*F*(1, 2412) = 17.41, *p* < 0.001, *η*^*2*^_*p*_ = 0.007); pet and non-pet owners who have either no children (*F*(1, 2412) = 2.15, *p* = 0.143, *η*^*2*^_*p*_ = 0.001), those who have 1 child living with them at home (*F*(1, 2412) = 3.46, *p* = 0.063, *η*^*2*^_*p*_ = 0.001), and those who have 2 children living with them at home (*F*(1, 2412) = 0.00, *p* = 0.987, *η*^*2*^_*p*_ = 0.000) did not differ in their levels of loneliness. Similarly, the significant interaction observed on stress reveals that pet owners who have 3 or more children living with them at home, as well as those with 2 children living at home reported higher stress compared to non-pet owners who have 3 or more children living with them at home (*F*(1, 2410) = 6.02, *p* = 0.014, *η*^*2*^_*p*_ = 0.002) and those who have 2 children living with them at home (*F*(1, 2410) = 4.88, *p* = 0.027, *η*^*2*^_*p*_ = 0.002). Pet and non-pet owners who have no children (*F*(1, 2410) = 0.00, *p* = 0.947, *η*^*2*^_*p*_ = 0.000), and those who have 1 child living with them at home (*F*(1, 2410) = 1.10, *p* = 0.294, *η*^*2*^_*p*_ = 0.000), did not differ in their levels of stress. The interaction effects uncovered here also align with a possible strain explanation, such that people who have 2 or more children at home may become burdened by the presence of a pet in the COVID context.

## Discussion

Whereas the popular media typically presents pets as beneficial to human health and mental health^6,7^, and as helping humans combat loneliness and stress during the COVID-19 pandemic^53,54^, previous research findings about the role played by pets in human health and well-being remain mixed^13,32^. To this aim, the current study explored for whom pet ownership can be more or less beneficial during a time of high stress. All statistical analyses included a poststratification weight to ensure the representativeness of the current sample relative to the general Canadian population, to account for potentially confounding sociodemographic factors, and to maximize the comparability of the pet and non-pet owner subgroups; applying the poststratification weight to the current data ensured that comparisons of pet vs. non-pet owners were not confounded by sociodemographic differences. A range of psychological well-being measures were taken: while some had been included in prior studies comparing pet and non-pet owners (e.g., stress, loneliness), additional measures capture more positive facets of well-being (e.g., vitality).

When comparing pet and non-pet owners in terms of their psychological well-being, we found that pet owners reported lower psychological well-being on four of the well-being measures (i.e., lower vitality, life satisfaction, presence of life meaning, but higher loneliness). Furthermore, pet owners reported experiencing more COVID-related impacts compared to non-pet owners. These findings suggest pet ownership during a stressful event like COVID may bring added stress to an already challenging context. Importantly, when accounting for the species of the pets participants owned (i.e., dog(s), cat(s), other pet species) and the number of pets, the majority of the effects uncovered for general pet ownership remained significant, and the same overall pattern of findings was observed. However, and in line with some prior research (e.g.,^55^), including recent research conducted during the COVID-19 pandemic^56^, dog ownership, compared to the ownership of other species of pets (i.e., including, but not restricted to, cats), was associated with higher well-being (i.e., higher vitality and life satisfaction; lower loneliness and COVID-related impacts) in this context (see also^57^, and^58^ on the negative effects of pet ownership being driven by cat ownership compared to dog ownership). These effects could be due to dogs’ particularly clear capacity to elicit physical outdoor activity and social interactions with fellow humans^59,60^, within the limits imposed during the pandemic.

To explore for whom pet ownership may impact on well-being the most, we examined differences between pet and non-pet owners drawing on sociodemographic data. Our findings were generally consistent with the notion that pet ownership may represent an added burden when personal or financial resources are already thin. First, pet ownership was associated with lower well-being (i.e., lower vitality, life satisfaction, presence of life meaning, higher loneliness) among women but not among men. This corresponds to research showing that women were impacted to a greater extent by the pandemic with increased childcare and housework responsibilities^61^. Second, pet ownership was associated with lower well-being (i.e., higher loneliness and stress) among people who have 2 children, and those who have 3 and more children, currently living at home with them. Again, this is consistent with research showing increased mental distress among parents with children during the pandemic^41,62^. Third, pet owners reported lower psychological well-being (i.e., lower life satisfaction, presence of life meaning) if they were unemployed or had more unstable forms of employment (e.g., students, homemakers). For these participants, the experience of pet ownership may represent an additional financial burden or responsibility.

The experience of negative COVID-related impacts provides a converging picture, with pet owners (vs. a non-pet owner) reporting higher COVID-related impacts if they were women, unemployed, and those with lower incomes. Beneficial effects of pet ownership in attenuating the experience of COVID-related impacts was found only among young adults (18–24 years-old), suggesting that those who benefited from pet ownership during the pandemic had fewer family-related responsibilities (e.g., child rearing).

Taken together, these findings suggest that occupying typically caring roles during the pandemic (e.g., having at least 2 children living at home; spending time and energy caring for children) and experiencing situations of disadvantage (i.e., being unemployed) provided a source of strain on available resources; by adding to these existing levels of strain, pet ownership was associated with reduced well-being. Emerging findings observed during the COVID-19 pandemic have also uncovered null and negative associations between the presence of pets and human wellness. For example, in a longitudinal study conducted among adolescents, Mueller, Richer, Callina, and Charmaraman^63^ found that pet ownership predicted higher levels of loneliness during COVID-19 as well as higher increases in loneliness from before to during the pandemic. Phillipou and colleagues^64^ found that pet ownership was significantly associated with poorer quality of life during the COVID-19 pandemic, but was not significantly associated with resilience or loneliness. In a large cross-sectional study, Ratschen and colleagues^45^ found that while pet ownership was associated with smaller decreases in mental health since lockdown, a majority of pet owners (67.6%) reported having been worried about their pet because of the pandemic (e.g., restricted access to veterinary care, restrictions to exercise/walks), suggesting this added responsibility in times of COVID can create an additional strain (see also^65^). The current findings build on this prior work by bringing additional nuance to our understanding of the role of pets during the pandemic – providing insights across a range of well-being measures and showing differential effects across segments of the population. Our findings, together with this prior research, suggest that our intuition that pets are good for well-being at times of high stress may be largely inaccurate.

The current findings have implications for decisions to adopt a pet during times of high strain like the COVID-19 pandemic, revealing that such decisions should take into account existing strain on personal and financial resources. While the effect sizes for our statistically significant effects (which ranged from *η*^*2*^_*p*_ = 0.002 to 0.005 in the analyses of variance) are considered of small magnitude^66^ and account for, at the most, approximately 0.05% of the variance in the prediction of the well-being measures, they do point to what appears to be a robust and generalizable trend. Our findings also call for a deeper reflection about pet ownership in the context of social inequalities and disadvantage^67,68^; our results indicate that in order to reap the benefits of pet ownership, people need sufficient personal and financial resources, and a lack of these resources may turn pet ownership into a burden for well-being at times of acute stress.

Given the methodological and statistical procedures employed, our findings can be generalized to the Canadian population and build on existing studies by surveying participants from diverse sociodemographic backgrounds (e.g., in terms of SES, gender; see^37,38^). The comparison of pet and non-pet owners was also robust due to our approach of statistically adjusting each of the two subgroups (i.e., pet and non-pet owners) to the overall Canadian population, therefore increasing the robustness of this specific comparison. Nevertheless, and given the cross-sectional nature of the current study, future research could employ longitudinal and/or experimental designs (using ethically sound procedures) to further test the direction of causality between pet ownership and human wellness. While the current study was not specifically designed (i.e., based on the quotas and poststratification weight variable employed) to examine the effects of the species of participants’ pets, future research should be designed to systematically and robustly test the impact of owning different species of pets on human wellness. Furthermore, future work should directly measure the quality of people’s relationships with their pets when assessing the benefits (or lack thereof) of pet ownership.

In sum, this research provided a systematic and nuanced account for the role played by pets in human well-being during the current COVID-19 pandemic. In light of the current depictions of pets as being beneficial to human wellness during this historic period, the present findings provide a counterpoint to these commonly shared assumptions. We hope that the rigorous methodological and statistical approaches employed herein, combined with a data-driven approach, will continue raising research interest in a widespread social phenomenon: the experience of pet ownership.

## Methods

### Recruitment

Results were based on a nationally representative survey (based on age, gender, region, and language) of 2424 Canadian adults (18 and older), conducted by the survey firm Léger from September 24 to October 7, 2020. Based on Canada’s total population (38 million), this sample size involves a margin of error of 2% and a 95% confidence level. Quotas were also imposed to recruit an equal number of pet and non-pet owners; the sample finally included 1220 pet owners and 1204 non-pet owners. Participants were invited to participate in the study via an email sent by Léger. All invitations were bilingual and participants could complete the questionnaire in either French or English, which are the two official languages in Canada. Respondents were drawn from Léger’s LEO internet panel, a widely used national probability-based online panel that includes over 420,000 households across Canada; it is the largest Canadian-owned panel. Within this panel, 61% of participants have been recruited randomly over the phone, 65% of the profiles have been updated in the last six months, and 50% of the profiles are based on the 2016 Statistics Canada census. Léger panels have been used in other peer-reviewed academic research^69–72^.

Léger administered the Qualtrics-based online questionnaire. The following measures were taken to maximize participation and the representativity of the final sample: the survey was open for two weeks, so as to leave sufficient time for participants to open the invitation email and complete the questionnaire; the email invites were sent in waves over this 2-week data collection period; participants could stop and continue the questionnaire at a later point in time, without loosing their responses; the questionnaire was accessible 24/7, via computer or mobile devises, so as to maximise participation among all age groups; in case of technical problems, participants could directly reach Léger’s technical support team by email or phone; email reminders were sent to nonresponders.

A total of 20,320 email invitations were sent to panel members, of which 3770 opened the invitation email. Among those, 96 refused to take part in the study, and 192 participants were considered non eligible (i.e., 72 did not consent to taking part in the study, 4 were non-eligible on the basis of their age, 3 lived outside of Canada, 113 failed one of the two attention check questions), and 670 had incomplete data (i.e., they did not reach the end of the questionnaire). This resulted in 2424 qualified completes used for analysis. When considering the total number of email invitations sent to potential participants, the participation rate is 12%; when not considering the individuals who have not opened the invitation email in this calculation, the participation rate is 64%. Median response time among qualified completes was 32 min. Participants were paid the equivalent of CAN$3 directly by Léger for participating in this study. Participants’ compensation takes the form of points that can be redeemed from different merchants (e.g., Starbucks, Tim Hortons) and entry into a draw (full details available via: www.legeropinion.com/fr/recompenses/). Informed consent is obtained from all the participants. The study was approved by the Ethics Committee involving Human Participants of the University of Québec in Montréal and was conducted in line with the Canadian Tri-Council Policy for the Ethical Conduct of Research Involving Humans.

### Poststratification weights

Poststratification statistical weights were prepared by Léger and provided to the research team, and then used in the main statistical analyses to account for differences between our sample and Canadian Census benchmarks. Based on the most recent data from Statistics Canada, the following benchmark distributions of Canadians who are 18 years and older from the general population were used to compute the poststratification weights: gender, age, Province of residence, native language, education, type of dwelling, marital status, area lived in (rural or urban), ethnicity, gross annual household income, employment status, presence of children in the household.

These sociodemographic variables were chosen on the basis of their utility for adjusting the current sample to the general Canadian population, and of recommendations for conducting research comparing pet and non-pet owners^19,32^. The poststratification weight variable used in the main analyses reported in the current study adjusts both the pet and non-pet owners subgroups to the general Canadian population on these sociodemographic variables. Doing so ensures that the poststratification weight variable is based on known data (Canadian Census), that both subgroups are each adjusted idiosyncratically to the Canadian population, and maximises these subgroups’ comparability.

### Questionnaire and measuring instruments

The measuring instruments were translated from English to French using a back-to-back translation procedure. When conducting this translation, the research assistants were instructed by the lead researcher to give priority to loyalty of meaning and familiarity of the content instead of strict loyalty to the original language (i.e., a decentering approach^73^). The individual measures included in the current study were taken from a larger representative survey pertaining to relationships with pets, close others, and well-being.

#### Sociodemographic and pet ownership information

The sociodemographic data included the following categories: gender (male, female, other), age (18–21, 22–24, 25–29, 30–34, 35–39, 40–44, 45–49, 50–54, 55–59, 60–64, 65–69, 70–74, > 75 years), education level (Primary school diploma, High school diploma, Diploma of Collegial studies (CEGEP), Professional studies diploma, Bachelor's degree, Master's degree, Doctoral degree, Other), dwelling type (apartment/condo, house, other), area lived in (city, suburb, countryside), annual household gross income (less than $20,000, $40,000–$59,999, $60,000–$79,999, $80,000–$99,999, $100,000–$119,999, $120,000–$139,999, $140,000–$159,999, $160,000–$179,999, $180,000–$199,999, over $200,000), employment (full-time, part-time, temporary, self-employed, unemployed, student, homemaker, retired), ethnicity (Caucasian or White, Native Canadian or Inuit, Metis, Black or African Canadian, East Asian or South-East Asian, South Asian, Middle Eastern or North African or Central Asian, Hispanic or Latino, other), marital status (common-law union, married, separated, divorced, single, widowed, single parent, other), number of children currently living at home. Participants were asked if they have lost their job during COVID (yes, no). To measure pet ownership, participants were asked: “Do you have one or more pet(s) currently?” (yes, no). To assess the number of pets currently owned, pet owners were asked: ‘‘How many pets do you currently have?’’ (non-pet owners were attributed a score of zero on this variable). To measure the species of their pet(s), pet owners were asked: “Please specify the species of this/these pet(s) you currently have” (bird, cat, dog, fish, reptile, rodent, other, please specify), for up to five pets.

#### COVID-related impacts

This measure is composed of three items: ‘To what extent are you experiencing stress as a result of the current COVID-19 epidemic?’; ‘To what extent are you experiencing uncertainty as a result of the current COVID-19 epidemic?’; ‘To what extent are you experiencing family-related issues as a result of the current COVID-19 epidemic?’ (see^74^). Participants provided their responses on a 1 (*no stress/uncertainty/family-related issues at all*) to 7 scale (*extreme stress/extreme uncertainty/a very large number of family-related issues*). This measure showed adequate reliability, as indicated by its satisfactory Cronbach's alpha (α) = 0.82. For this measure and each of the psychological well-being measures presented below, composite scores were created by calculating the mean of the measure’s items.

#### Psychological well-being measures

A broad range of well-being measures were included, in line with a holistic conception of human health and wellness^75^. These measures had been selected on the basis of their validity and established psychometric properties. In addition to providing the standard instructions for completing each scale, each measure asked participants to respond by referring specifically to how they feel during the COVID-19 pandemic.

*Vitality* was evaluated with seven items^76^. This scale measures the energizing aspect of psychological well-being (e.g., ‘I feel alive and vital’; *α* = 0.92). Responses were made on a 1 (*does not correspond at all*) to 7 (*corresponds exactly*) scale. *Loneliness* was assessed using the UCLA Loneliness Scale (Version 3)^77^. This 20-item scale is designed to measure one’s subjective feelings of loneliness as well as feelings of social isolation (e.g., ‘How often do you feel that you are “in tune” with the people around you?’; *α* = 0.93). Participants rated each item on a scale from 1 (*never*) to 4 (*always*). *Life satisfaction* was measured with the 5-item Satisfaction With Life Scale^78^ (e.g., ‘In most ways my life is close to my ideal’; *α* = 0.91). Participants indicated their agreement with each item using a 1 (*strongly disagree*) to 7 (*Strongly agree*) scale. To assess *presence of life meaning* (*α* = 0.90)^79^, participants were asked to think about what makes their life feel important to them and respond to 5 items as truthfully and accurately as they could on a scale from 1 (*absolutely untrue*) to 7 (*absolutely true*); higher scores are indicative of higher psychological well-being^80^ (e.g., ‘My life has a clear sense of purpose’). The 14-item *Perceived Stress Scale*^81^ assesses the degree to which people perceive their lives as stressful (e.g., ‘How often have you been upset because of something that happened unexpectedly?’; *α* = 0.87). Participants rated each item on a scale from 1 (*never*) to 5 (*very often*).

### Statistical analyses

Statistical analyses were performed using SPSS (IBM SPSS Statistics for Windows, Version 27.0. Armonk, NY: IBM Corp.). Table 1 presents the results of the analyses of variance (ANOVAs) conducted to compare pet and non-pet owners on the psychological well-being measures; the data used for these analyses are weighted. The hierarchical regression analyses reported in the text also included weighted data. Tables 2, 3, 4, 5, 6, 7 and 8 present the results of the ANOVAs conducted to compare pet and non-pet owners’ well-being at the different levels of the sociodemographic variables; the data used for these analyses are also weighted.

## Supplementary Information

Supplementary Information.

## Data Availability

The data and codes that support the findings presented in this manuscript can be accessed, for verification purposes only, via the following link: https://osf.io/56sbh/?view_only=bbca7561fdff46618666454a0d66c892.
