# Supplementary material for: Pet ownership and psychological well-being during the COVID-19 pandemic
Source: Sci Rep. 2022 Apr 12;12:6091. doi: 10.1038/s41598-022-10019-z (PMC9002031; doi:10.1038/s41598-022-10019-z)
Supplement: Supplementary file 1 — Supplementary Information. [file 41598_2022_10019_MOESM1_ESM.docx]

Supplementary Information for:

**Pet Ownership and Psychological Well-Being during the COVID-19 Pandemic: A Representative Study Conducted in Canada**

Catherine E. Amiot^1^, Christophe Gagné^1^, and Brock Bastian^2^

^1^ Département de psychologie, Université du Québec à Montréal, C.P. 8888, Succ. Centre-Ville, Montréal, PQ, H3C 3P8, Canada

^2^ Melbourne School of Psychological Sciences, The University of Melbourne, 06, 05, Redmond Barry Building, Parkville, VIC 3010, Australia

* E-mail: amiot.catherine@uqam.ca, Tel.: (514) 987-3000, ext. 5006, Fax: (514) 987-7953

This supplementary information document includes Tables S1, S2, S3, S4, and S5, the information and consent form, as well as the questionnaire measures employed in the current study.

Table S1

*Sociodemographic Information for the Overall Sample and for Pet and Non-Pet Owners (Unweighted Data).*

|  |  | | Overall Sample | | Pet Owners (*N*=1220) | | | Non-Pet Owners (*N*=1204) | | |  | |
| --- | --- | --- | --- | --- | --- | --- | --- | --- | --- | --- | --- | --- |
|  |  | | *N* | % | | *N* | % | | *N* | % | | Statistics |
| Gender | Male | | 1200 | 49.5 | | 542 | 44.4^a^ | | 658 | 54.7^a^ | | X^2^ (2) = 25.65  *p* < .001 |
|  | Female | | 1218 | 50.2 | | 674 | 55.2^a^ | | 544 | 45.2^a^ | |  |
|  | Other | | 6 | 0.2 | | 4 | 0.3 | | 2 | 0.2 | |  |
|  |  | | *N* | % | | *N* | % | | *N* | % | |  |
| Age | 18 to 21 years old | | 122 | 5.0 | | 61 | 5.0 | | 61 | 5.1 | | X^2^ (12) = 68.67  *p* < .001 |
|  | 22 to 24 years old | | 129 | 5.3 | | 73 | 6.0 | | 56 | 4.7 | |  |
|  | 25 to 29 years old | | 169 | 7.0 | | 89 | 7.3 | | 80 | 6.6 | |  |
|  | 30 to 34 years old | | 198 | 8.2 | | 104 | 8.5 | | 94 | 7.8 | |  |
|  | 35 to 39 years old | | 222 | 9.2 | | 127 | 10.4^a^ | | 95 | 7.9^a^ | |  |
|  | 40 to 44 years old | | 201 | 8.3 | | 100 | 8.2 | | 101 | 8.4 | |  |
|  | 45 to 49 years old | | 230 | 9.5 | | 128 | 10.5 | | 102 | 8.5 | |  |
|  | 50 to 54 years old | | 283 | 11.7 | | 162 | 13.3^a^ | | 121 | 10.0^a^ | |  |
|  | 55 to 59 years old | | 217 | 9.0 | | 110 | 9.0 | | 107 | 8.9 | |  |
|  | 60 to 64 years old | | 220 | 9.1 | | 119 | 9.8 | | 101 | 8.4 | |  |
|  | 65 to 69 years old | | 207 | 8.5 | | 77 | 6.3^a^ | | 130 | 10.8^a^ | |  |
|  | 70 to 74 years old | | 137 | 5.7 | | 50 | 4.1^a^ | | 87 | 7.2^a^ | |  |
|  | 75 or older | | 89 | 3.7 | | 20 | 1.6^a^ | | 69 | 5.7^a^ | |  |
|  |  | | *N* | % | | *N* | % | | *N* | % | |  |
| Language of Questionnaire | English | | 2006 | 82.8 | | 974 | 79.8^a^ | | 1032 | 85.7^a^ | | X^2^ (1) = 14.67  *p* < .001 |
|  | French | | 418 | 17.2 | | 246 | 20.2^a^ | | 172 | 14.3^a^ | |  |
|  |  | | *N* | % | | *N* | % | | *N* | % | |  |
| Education | Primary school diploma | | 37 | 1.5 | | 22 | 1.8 | | 15 | 1.2 | | X^2^ (8) = 29.78  *p* < .001 |
|  | High school diploma | | 539 | 22.2 | | 283 | 23.2 | | 256 | 21.3 | |  |
|  | Diploma of Collegial studies (CEGEP) | | 483 | 19.9 | | 279 | 22.9^a^ | | 204 | 16.9^a^ | |  |
|  | Professional studies diploma | | 217 | 9.0 | | 116 | 9.5 | | 101 | 8.4 | |  |
|  | Bachelor's degree | | 814 | 33.6 | | 379 | 31.1^a^ | | 435 | 36.1^a^ | |  |
|  | Master's degree | | 260 | 10.7 | | 107 | 8.8^a^ | | 153 | 12.7^a^ | |  |
|  | Doctoral degree | | 39 | 1.6 | | 15 | 1.2 | | 24 | 2.0 | |  |
|  | Other | | 32 | 1.3 | | 17 | 1.4 | | 15 | 1.2 | |  |
|  | Did not answer | | 3 | 0.1 | | 2 | 0.2 | | 1 | 0.1 | |  |
|  |  | | *N* | *%* | | *N* | *%* | | *N* | *%* | |  |
| Dwelling Type | Apartment/Condo | | 761 | 31.4 | | 295 | 24.2^a^ | | 466 | 38.7^a^ | | X^2^ (2) = 60.36  *p* < .001 |
|  | House | | 1637 | 67.5 | | 908 | 74.4^a^ | | 729 | 60.5^a^ | |  |
|  | Other | | 26 | 1.1 | | 17 | 1.4 | | 9 | 0.7 | |  |
|  |  | | *N* | % | | *N* | % | | *N* | % | |  |
| Area | City | | 1236 | 51.0 | | 566 | 46.4^a^ | | 670 | 55.6^a^ | | X^2^ (2) = 37.47  *p* < .001 |
|  | Suburb | | 848 | 35.0 | | 435 | 35.7 | | 413 | 34.3 | |  |
|  | Countryside | | 340 | 14.0 | | 219 | 18.0^a^ | | 121 | 10.0^a^ | |  |
|  |  | | *N* | % | | *N* | % | | *N* | % | |  |
| Annual Household Gross Income | Less than $20,000 | | 161 | 6.6 | | 75 | 6.1 | | 86 | 7.1 | | X^2^ (11) = 14.83  *p* = .190 |
|  | $20,000 to $39,999 | | 328 | 13.5 | | 166 | 13.6 | | 162 | 13.5 | |  |
|  | $40,000 to $59,999 | | 369 | 15.2 | | 166 | 13.6^a^ | | 203 | 16.9^a^ | |  |
|  | $60,000 to $79,999 | | 330 | 13.6 | | 169 | 13.9 | | 161 | 13.4 | |  |
|  | $80,000 to $99,999 | | 322 | 13.3 | | 167 | 13.7 | | 155 | 12.9 | |  |
|  | $100,000 to $119,999 | | 240 | 9.9 | | 130 | 10.7 | | 110 | 9.1 | |  |
|  | $120,000 to $139,999 | | 136 | 5.6 | | 77 | 6.3 | | 59 | 4.9 | |  |
|  | $140,000 to $159,999 | | 122 | 5.0 | | 62 | 5.1 | | 60 | 5.0 | |  |
|  | $160,000 to $179,999 | | 63 | 2.6 | | 38 | 3.1 | | 25 | 2.1 | |  |
|  | $180,000 to $199,999 | | 53 | 2.2 | | 26 | 2.1 | | 27 | 2.2 | |  |
|  | Over $200,000 | | 89 | 3.7 | | 49 | 4.0 | | 40 | 3.3 | |  |
|  | Prefer not to answer | | 211 | 8.7 | | 95 | 7.8 | | 116 | 9.6 | |  |
|  |  | | *N* | % | | *N* | % | | *N* | % | |  |
| Employment | Full-time | | 1092 | 45.0 | | 581 | 47.6^a^ | | 511 | 42.4^a^ | | X^2^ (7) = 34.66  *p* < .001 |
|  | Part-time | | 204 | 8.4 | | 109 | 8.9 | | 95 | 7.9 | |  |
|  | Temporary | | 8 | 0.3 | | 5 | 0.4 | | 3 | 0.2 | |  |
|  | Self-employed | | 158 | 6.5 | | 84 | 6.9 | | 74 | 6.1 | |  |
|  | Unemployed | | 180 | 7.4 | | 92 | 7.5 | | 88 | 7.3 | |  |
|  | Student | | 158 | 6.5 | | 85 | 7.0 | | 73 | 6.1 | |  |
|  | Homemaker | | 95 | 3.9 | | 56 | 4.6 | | 39 | 3.2 | |  |
|  | Retired | | 529 | 21.8 | | 208 | 17.0^a^ | | 321 | 26.7^a^ | |  |
|  |  | | *N* | % | | *N* | % | | *N* | % | |  |
| Ethnicity | Caucasian or White | | 1865 | 76.9 | | 1017 | 83.4^a^ | | 848 | 70.4^a^ | | X^2^ (9) = 79.05  *p* < .001 |
|  | Native Canadian or Inuit | | 30 | 1.2 | | 13 | 1.1 | | 17 | 1.4 | |  |
|  | Metis | | 10 | 0.4 | | 6 | 0.5 | | 4 | 0.3 | |  |
|  | Black or African Canadian | | 60 | 2.5 | | 17 | 1.4^a^ | | 43 | 3.6^a^ | |  |
|  | East Asian or South-East Asian | | 239 | 9.9 | | 79 | 6.5^a^ | | 160 | 13.3^a^ | |  |
|  | South Asian | | 107 | 4.4 | | 32 | 2.6^a^ | | 75 | 6.2^a^ | |  |
|  | Middle Eastern, North African, or Central Asian | | 34 | 1.4 | | 11 | 0.9^a^ | | 23 | 1.9^a^ | |  |
|  | Hispanic or Latino | | 29 | 1.2 | | 16 | 1.3 | | 13 | 1.1 | |  |
|  | Other | | 45 | 1.9 | | 25 | 2.0 | | 20 | 1.7 | |  |
|  | Prefer not to answer | | 5 | 0.2 | | 4 | 0.3 | | 1 | 0.1 | |  |
|  |  | | *N* | % | | *N* | % | | *N* | % | |  |
| Marital Status | Common-law union | | 364 | 15.0 | | 236 | 19.3^a^ | | 128 | 10.6^a^ | | X^2^ (8) = 50.46  *p* < .001 |
|  | Married | | 1097 | 45.3 | | 562 | 46.1 | | 535 | 44.4 | |  |
|  | Separated | | 68 | 2.8 | | 35 | 2.9 | | 33 | 2.7 | |  |
|  | Divorced | | 158 | 6.5 | | 65 | 5.3^a^ | | 93 | 7.7^a^ | |  |
|  | Single | | 657 | 27.1 | | 283 | 23.2^a^ | | 374 | 31.1^a^ | |  |
|  | Widowed | | 61 | 2.5 | | 29 | 2.4 | | 32 | 2.7 | |  |
|  | Single parent | | 13 | 0.5 | | 7 | 0.6 | | 6 | 0.5 | |  |
|  | Other | | 2 | 0.1 | | 1 | 0.1 | | 1 | 0.1 | |  |
|  | Prefer not to answer | | 4 | 0.2 | | 2 | 0.2 | | 2 | 0.2 | |  |
|  |  | | *M* | *SD* | | *M* | *SD* | | *M* | *SD* | |  |
| Number of Children Currently Living at Home | | | 0.95 | 1.01 | | 1.07 | 0.98 | | 0.82 | 1.02 | | *t* (1340) = -0.27  *p* = .784 |
|  | | | *N* | % | | *N* | % | | *N* | % | |  |
| Job Lost in COVID-19 | | Yes | 359 | 14.8 | | 184 | 15.1 | | 175 | 14.5 | | X^2^ (2) = 3.95  *p* = .139 |
|  |  | No | 2032 | 83.8 | | 1025 | 84 | | 1007 | 83.6 | |  |
|  |  | Prefer not to answer | 33 | 1.4 | | 11 | 0.9 | | 22 | 1.8 | |  |

*Notes.* Within a row, means with the same superscript differ in the Wald z-test analyses (2-tailed) comparing pet and non-pet owners at each level of the demographic variable (*p* < .05)

Table S2

*Results of ANOVAs Comparing Pet and Non-Pet Owners Across Ethnicities on the Well-Being and COVID-Related Impacts Variables (Weighted Data).*

|  | *Pet Owners* | | | | *Non-Pet Owners* | | | | *Pet Ownership* | | *Ethnicity* | | *Pet Ownership x Ethnicity* | |
| --- | --- | --- | --- | --- | --- | --- | --- | --- | --- | --- | --- | --- | --- | --- |
|  | *White* | | *Other Ethnicities* | | *White* | | *Other Ethnicities* | |  | |  | |  | |
|  | *M* | *SD* | *M* | *SD* | *M* | *SD* | *M* | *SD* | *F* | *η^2^_p_* | *F* | *η^2^_p_* | *F* | *η^2^_p_* |
| Vitality | 4.24 | 1.28 | 4.22 | 1.53 | 4.32 | 1.30 | 4.41 | 1.25 | 4.46* | .002 | 0.36 | .000 | 0.80 | .000 |
| Loneliness | 2.25 | 0.51 | 2.41^a^ | 0.66 | 2.20 | 0.54 | 2.30^a^ | 0.46 | 8.27** | .003 | 26.51*** | .01 | 1.26 | .001 |
| Life Satisfaction | 4.40^a^ | 1.39 | 4.33 | 1.78 | 4.60^a^ | 1.41 | 4.37 | 1.28 | 3.01 | .001 | 4.73* | .002 | 1.40 | .001 |
| Presence of Life Meaning | 4.62^a^ | 1.32 | 4.45 | 1.57 | 4.76^a^ | 1.30 | 4.67 | 1.15 | 7.97** | .003 | 4.16* | .002 | 0.33 | .000 |
| Stress | 2.75^a^ | 0.60 | 2.89 | 0.69 | 2.68^a^ | 0.61 | 2.92 | 0.44 | 0.56 | .000 | 43.08*** | .018 | 2.85 | .001 |
| COVID-Related Impacts | 3.52^a^ | 1.20 | 3.86 | 1.88 | 3.29^a^ | 1.19 | 3.89 | 1.25 | 2.72 | .001 | 58.75*** | .024 | 4.61* | .002 |

*Notes.* ^*^*p* < .05; ^**^*p* < .01; ^***^*p* < .001. The ‘White’ ethnicity category included Whites and Caucasians (*n*=1865); the ‘Other Ethnicities’ category included all other ethnic groups (*n*=509). These two categories were used given the particularly uneven cell sizes observed for the specific ethnic groups (please see Table S1), and based on the American Psychological Association recommendations for referring to ethnic groups (https://apastyle.apa.org/style-grammar-guidelines/bias-free-language/racial-ethnic-minorities). Within a row, means with the same superscript differ in the paired comparison analyses comparing pet and non-pet owners at each level of the demographic variable (*p* < .05)

Table S3

*Results of ANOVAs Comparing Pet and Non-Pet Owners Across Education Levels on the Well-Being and COVID-Related Impacts Variables (Weighted Data).*

|  | *Pet Owners* | | | | *Non-Pet Owners* | | | | *Pet Ownership* | | *Education* | | *Pet Ownership x Education* | |
| --- | --- | --- | --- | --- | --- | --- | --- | --- | --- | --- | --- | --- | --- | --- |
|  | *Pre-University Diploma* | | *University Diploma* | | *Pre-University Diploma* | | *University Diploma* | |  | |  | |  | |
|  | *M* | *SD* | *M* | *SD* | *M* | *SD* | *M* | *SD* | *F* | *η^2^_p_* | *F* | *η^2^_p_* | *F* | *η^2^_p_* |
| Vitality | 4.17^a^ | 1.52 | 4.33 | 0.97 | 4.35^a^ | 1.57 | 4.29 | 0.90 | 1.34 | .001 | 0.81 | .000 | 3.24 | .001 |
| Loneliness | 2.31^a^ | 0.62 | 2.25 | 0.42 | 2.23^a^ | 0.64 | 2.22 | 0.36 | 5.69* | .002 | 2.55 | .001 | 1.49 | .001 |
| Life Satisfaction | 4.22^a^ | 1.65 | 4.66 | 1.07 | 4.48^a^ | 1.70 | 4.70 | 0.99 | 4.90* | .002 | 25.90*** | .011 | 3.04 | .001 |
| Presence of Life Meaning | 4.52^a^ | 1.50 | 4.69 | 1.10 | 4.71^a^ | 1.54 | 4.85 | 0.87 | 8.63** | .004 | 7.22** | .003 | 0.07 | .000 |
| Stress | 2.81^a^ | 0.70 | 2.75 | 0.46 | 2.73^a^ | 0.69 | 2.76 | 0.42 | 1.53 | .001 | 0.29 | .000 | 2.71 | .001 |
| COVID-Related Impacts | 3.60^a^ | 1.50 | 3.64 | 1.02 | 3.35^a^ | 1.49 | 3.66 | 0.90 | 3.92* | .002 | 8.47** | .004 | 5.60* | .002 |

*Notes.* ^*^*p* < .05; ^**^*p* < .01; ^***^*p* < .001. The Pre-University Diploma category included participants who had obtained a primary school diploma, a high school diploma, a college degree, or a professional studies diploma (*n*=1276); the University Diploma category included participants who had completed a bachelor’s, master’s or doctoral degree (*n*=1113). These two education categories were used in the analysis given the uneven cell sizes observed for the specific education levels (Table S1), and given their use in the post-stratification weight variable. Within a row, means with the same superscript differ in the paired comparison analyses comparing pet and non-pet owners at each level of the demographic variable (*p* < .05).

Table S4

|  | *Pet Owners* | | | | *Non-Pet Owners* | | | | *Pet Ownership* | | *Martial Status* | | *Pet Ownership x Marital Status* | |
| --- | --- | --- | --- | --- | --- | --- | --- | --- | --- | --- | --- | --- | --- | --- |
|  | *Married/*  *Common Law* | | *Other Statuses* | | *Married/*  *Common Law* | | *Other Statuses* | |  | |  | |  | |
|  | *M* | *SD* | *M* | *SD* | *M* | *SD* | *M* | *SD* | *F* | *η^2^_p_* | *F* | *η^2^_p_* | *F* | *η^2^_p_* |
| Vitality | 4.42 | 1.14 | 4.00^a^ | 1.59 | 4.47 | 1.16 | 4.20^a^ | 1.40 | 5.69* | .002 | 42.96*** | .017 | 2.09 | .001 |
| Loneliness | 2.19^a^ | 0.47 | 2.40^b^ | 0.64 | 2.13^a^ | 0.45 | 2.33^b^ | 0.57 | 9.48** | .004 | 98.00*** | .039 | 0.06 | .000 |
| Life Satisfaction | 4.68^a^ | 1.21 | 4.00^b^ | 1.74 | 4.87^a^ | 1.16 | 4.17^b^ | 1.53 | 10.51** | .004 | 150.75*** | .059 | 0.06 | .000 |
| Presence of Life Meaning | 4.71^a^ | 1.16 | 4.41 | 1.65 | 5.01^a^ | 1.08 | 4.47 | 1.38 | 11.06*** | .005 | 63.45*** | .026 | 4.99* | .002 |
| Stress | 2.72 | 0.53 | 2.87 | 0.75 | 2.68 | 0.54 | 2.81 | 0.60 | 3.84 | .002 | 32.63*** | .013 | 0.36 | .000 |
| COVID-Related Impacts | 3.45 | 1.15 | 3.75 ^a^ | 1.58 | 3.35 | 1.15 | 3.54 ^a^ | 1.32 | 9.43** | .004 | 21.96*** | .009 | 1.23 | .001 |

*Results of ANOVAs Comparing Pet and Non-Pet Owners Across Marital Status on the Well-Being and COVID-Related Impacts Variables (Weighted Data).*

*Notes.* ^*^*p* < .05; ^**^*p* < .01; ^***^*p* < .001. The Married/Common Law category included participants who were married or in a common law union (*n*=1461); the Other Statuses category included all other civil status groups (*n*=963). These two marital categories were also used in the post-stratification weight variable. Within a row, means with the same superscript differ in the paired comparison analyses comparing pet and non-pet owners at each level of the demographic variable (*p* < .05).

Table S5

*Dog Owners and Cat Owners* *on the Well-Being and COVID-Related Impacts Variables (Weighted Data).*

|  | Dog Owners  *n*=690 | | Cat Owners  *n*=658 | |
| --- | --- | --- | --- | --- |
|  | *M* | *SD* | *M* | *SD* |
| Vitality | 4.38 | 1.30 | 4.09 | 1.31 |
| Loneliness | 2.24 | 0.53 | 2.33 | 0.53 |
| Life Satisfaction | 4.50 | 1.36 | 4.24 | 1.49 |
| Presence of Life Meaning | 4.64 | 1.34 | 4.52 | 1.37 |
| Stress | 2.77 | 0.61 | 2.82 | 0.62 |
| COVID-Related Impacts | 3.49 | 1.28 | 3.61 | 1.31 |

*Notes.* The dog owners category include participants who own at least one dog. The cat owners category include participants who own at least one cat.

**Information and Consent Form**

You are invited to take part in a research project that aims to study the nature of people’s relationships with pets and with close others during the COVID-19 pandemic as well as how people generally feel in their lives. We also aim to test the associations that may possibly exist between people’s relationships with pets and with close others as well as their level of psychological well-being during this unique time period. Before accepting to participate in this study, please take the time required to read the following information. If you have any question about the information contained in this consent form, please do not hesitate to contact us.

IDENTIFICATION: (Information of this section has been removed to ensure double-blind review)

FUNDING: This study is funded by the Social Sciences and Humanities Research Council of Canada (SSHRC).

PROCEDURES OR TASKS REQUESTED FROM PARTICIPANTS: Your participation involves completing a 30-minute online questionnaire. You will be invited to answer different questions concerning the relationship you have with pets and with close others as well as questions about your life in general. More specifically, the questionnaire involves completing scales that measure your connection to animals in general, your perceptions of the support provided by pets and by close others during the COVID-19 pandemic, your feelings and emotions experienced in the presence of pets and people who are close to you during this period, as well as the extent to which you feel connected to your community and motivated to participate in a variety of social activities. Finally, we will assess your general feelings of well-being felt during the pandemic. Please answer this questionnaire on a computer. This ensures the quality of the information

BENEFITS AND RISKS: Your participation will contribute to a better understanding of the factors related to our relationship with pets and with people during an unprecedented period in our history. This represents a major advancement in the field of social psychology, since to date, only a few studies have examined the respective roles of these relationships, which are very present in human life, when people are experiencing uncertainty and stress. There is no risk of significant discomfort associated with your participation. Please keep in mind when completing the questionnaire that there are no right or wrong answers; we are interested in how you actually think and feel. However, you must be aware that some questions may rekindle some unpleasant emotions. Please keep in mind that you remain free not to answer a question that may cause you discomfort without having to justify yourself. You can also stop to participate in the study at any time. Support resources are provided at the end of the study.

ANONYMITY AND CONFIDENTIALITY: All information collected in the questionnaire will remain confidential. The data obtained for this study will be kept in a secured database and the online questionnaire is hosted on a secure server. Only (information removed to ensure double-blind review), and the research assistant(s) involved in this study will have access to the data. Data will be analysed globally and it will not be possible to identify a specific participant. The data gathered for this study will be published in scientific journals, for scientific and educational purposes only. In keeping with best practices of open and transparent scientific research, we ask your permission to share some of the data you have provided with other researchers. Specifically, the computerized databases may be made available online to other researchers, but only for data verification purposes. However, these databases will not contain any identifying information (e.g., email).

VOLUNTARY PARTICIPATION: Your participation in this study is entirely voluntary. This means that you agree to participate in the study without any constraint or external pressure. Moreover, you are free to terminate your participation at any time during the study, without prejudice of any nature and without having to justify yourself. Your agreement to participate in this study also means that you agree that the research team may use the information collected for scientific and educational purposes (e.g., articles, theses of student team members, conferences and scientific papers) on the condition that no information that identifies you is publicly disclosed unless your explicit consent is obtained. Please note that we will not be able to destroy the questionnaire after submission since all data are anonymous.

CLAUSE OF LIABILITY: By agreeing to participate in this project, you do not waive any of your rights or release the researchers, the sponsor or the institutions involved from their legal and professional obligations. At the end of this project, we would like to retain your data for an unlimited period of time to be able to conduct other research projects. This involves analyzing your current data according to new research hypotheses. The ethical rules of this project apply to the long-term preservation of your data. You are free to refuse the secondary use of the data.

- I agree that my data be used for future research projects.
- I refuse that my data be used for future research projects.

QUESTIONS ABOUT THE PROJECT OR YOUR RIGHTS? (Information of this section has been removed to ensure double-blind review)

CONSENT:

Hereby:

a) I have read this information and consent form;

b) I voluntarily consent to participate in this research project;

c) I understand the goals of the project and what my participation implies;

d) I confirm that I have had sufficient time to consider my decision to participate in this study;

e) I also recognize that the project manager, and (the) research team have answered my questions satisfactorily; and

f) I understand that my participation in this research is completely voluntary and that I can withdraw from the study at any time without any penalties and without having to justify myself.

If you agree to participate in this study, please indicate your consent by choosing the "Yes, I consent to participate" answer below, which will direct you to the online questionnaire.

- Yes, I consent to participate.
- No

Questionnaire

[age] What is your age?

[r1] Under 18 years old

[r2] 18 to 21 years old

[r3] 22 to 24 years old

[r4] 25 to 29 years old

[r5] 30 to 34 years old

[r6] 35 to 39 years old

[r7] 40 to 44 years old

[r8] 45 to 49 years old

[r9] 50 to 54 years old

[r10] 55 to 59 years old

[r11] 60 to 64 years old

[r12] 65 to 69 years old

[r13] 70 to 74 years old

[r14] 75 or older

[gender] What is your gender?

[r1] Male

[r2] Female

[r96] Other, please specify:

[ethn] What is your ethnicity?

[r1] Caucasian or White (e.g., European, Russian background)

[r2] Native Canadian or Inuit

[r3] Metis

[r4] Black or African Canadian (e.g., Nigerian, Haitian)

[r5] East Asian or South-East Asian (e.g., Chinese, Vietnamese)

[r6] South Asian (e.g., Indian, Pakistani)

[r7] Middle Eastern, North African, or Central Asian (e.g., Saudi, Egyptian, Iranian)

[r8] Hispanic or Latino (e.g., Brazilian, Mexican, Cuban)

[r96] Other (please specify):

[Covid_stress] To what extent are you experiencing stress as a result of the current COVID-19 epidemic?

[r1] No stress at all

[r2] Very little stress

[r3] Little stress

[r4] Moderate stress

[r5] Quite a bit of stress

[r6] A lot of stress

[r7] Extreme stress

[r99] I prefer not to answer

[Covid_incerti] To what extent are you experiencing uncertainty as a result of the current COVID-19 epidemic?

[r1] No uncertainty at all

[r2] Very little uncertainty

[r3] Little uncertainty

[r4] Moderate uncertainty

[r5] Quite a bit of uncertainty

[r6] A lot of uncertainty

[r7] Extreme uncertainty

[r99] I prefer not to answer

[Covid_family] To what extent are you experiencing family-related issues as a result of the current COVID-19 epidemic?

[r1] No family-related issues at all

[r2] Very few family-related issues

[r3] A few family-related issues

[r4] A moderate number of family-related issues

[r5] Quite a few family-related issues

[r6] A lot of family-related issues

[r7] A very large number of family-related issues

[r99] I prefer not to answer

[AREA] In what area do you live?

[r1] City

[r2] Suburb

[r3] Countryside

[DWELLING] What type of dwelling do you live in?

[r1] Apartment/Condo

[r2] House

[r96] Other, please specify:

[MartialStatus] What is your marital status?

[r1] Common-law union

[r2] Married

[r3] Separated

[r4] Divorced

[r5] Single

[r6] Widowed

[r7] Single parent

[r96] Other, please specify:

[CHILD] Do you have one or more children?

[r1] Yes

[r2] No

[NumChild] How many children do you have?

[childWithYou] How many of your children currently live with you?

[workArrang] Please indicate your current work arrangements:

[r1] Full-time

[r2] Part-time

[r3] Temporary

[r4] Self-employed

[r5] Unemployed

[r6] Student

[r7] Homemaker

[r8] Retired

[Covid_loss] Have you lost your job in the current COVID-19 epidemic?

[r1] Yes

[r2] No

[r99] Prefer not to answer

[workHours] How many hours do you work each week (on average)?

[EDUC] What is the highest degree/qualification that you have earned?

[r1] Primary school diploma

[r2] High school diploma

[r3] Diploma of Collegial studies (CÉGEP)

[r4] Professional studies diploma

[r5] Bachelor’s degree

[r6] Master’s degree

[r7] Doctoral degree

[r96] Other, please specify:

[INCOME] Please indicate your gross annual household income:

[r1] Less than $20,000

[r2] $20, 000 to $39, 999

[r3] $40, 000 to $59, 999

[r4] $60, 000 to $79, 999

[r5] $80, 000 to $99, 999

[r6] $100, 000 to $119, 999

[r7] $120, 000 to $139, 999

[r8] $140, 000 to $159, 999

[r9] $160, 000 to $179, 999

[r10] $180, 000 to $199, 999

[r11] Over $200, 000

[r99] I prefer not to answer

[petOwn] Do you have one or more pet(s) currently?

[r1] Yes

[r2] No

[numPet] How many pets do you currently have?

Please specify the species of this/these pet(s) you currently have:

[Species1] Species of pet 1:

[r1] Bird

[r2] Cat

[r3] Dog

[r4] Fish

[r5] Reptile

[r6] Rodent

[r7] Other, please specify

[Species2] Species of pet 2:

[r1] Bird

[r2] Cat

[r3] Dog

[r4] Fish

[r5] Reptile

[r6] Rodent

[r7] Other, please specify

[Species3] Species of pet 3:

[r1] Bird

[r2] Cat

[r3] Dog

[r4] Fish

[r5] Reptile

[r6] Rodent

[r7] Other, please specify

[Species4] Species of pet 4:

[r1] Bird

[r2] Cat

[r3] Dog

[r4] Fish

[r5] Reptile

[r6] Rodent

[r7] Other, please specify

**[Species5] Species of pet 5:**

[r1] Bird

[r2] Cat

[r3] Dog

[r4] Fish

[r5] Reptile

[r6] Rodent

[r7] Other, please specify

For how long have you had this/these pet(s)? (in years)

[timePet1] Pet 1:

[timePet2] Pet 2:

[timePet3] Pet 3:

[timePet4] Pet 4:

[timePet5] Pet 5:

**[vital] Subjective vitality** [76]

*Responses were recorded on a scale from 1 (Not at all true) to 7 (Very true)*

For the following questions, please refer to yourself and your life in general. Using the scale below, please indicate how the following items correspond to how you feel during the COVID-19 pandemic:

[vital1] I feel alive and vital

[vital2] I don't feel very energetic

[vital3] Sometimes I feel so alive I just want to burst

[vital4] I have energy and spirit

[vital5] I look forward to each new day

[vital6] I nearly always feel alert and awake

[vital7] I feel energized

[lone] UCLA Loneliness Scale (version 3) [77]

The following statements describe how people sometimes feel. For each statement, please indicate how often you feel the way described using the following rating scale during the COVID-19 pandemic.

*Responses were recorded on a scale from 1 (Never) to 4 (Always)*

[lone1] How often do you feel that you are “in tune” with the people around you?

[lone2] How often do you feel that you lack companionship?

[lone3] How often do you feel that there is no one you can turn to?

[lone4] How often do you feel alone?

[lone5] How often do you feel part of a group of friends?

[lone6] How often do you feel that you have a lot in common with the people around you?

[lone7] How often do you feel that you are no longer close to anyone?

[lone8] How often do you feel that your interests and ideas are not shared by those around you?

[lone9] How often do you feel outgoing and friendly?

[lone10] How often do you feel close to people?

[lone11] How often do you feel left out?

[lone12] How often do you feel that your relationships with others are not meaningful?

[lone13] How often do you feel that no one really knows you well?

[lone14] How often do you feel isolated from others?

[lone15] How often do you feel that you can find companionship when you want it?

[lone16] How often do you feel that there are people who really understand you?

[lone17] How often do you feel shy?

[lone18] How often do you feel that people are around you but not with you?

[lone19] How often do you feel that there are people you can talk to?

[lone20] How often do you feel that there are people you can turn to?

[LifeSat] Satisfaction With Life Scale [78]

Below are five statements with which you may agree or disagree. Please indicate your level of agreement with each item by referring to how you feel specifically during the COVID-19 pandemic. Please be open and honest in your responding.

*Responses were recorded on a scale from 1 (Strongly disagree) to 7 (Strongly agree)*

[Lifesat1] In most ways my life is close to my ideal.

[Lifesat2] The conditions of my life are excellent.

[Lifesat3] I am satisfied with my life.

[Lifesat4] So far I have gotten the important things I want in life.

[Lifesat5] If I could live my life over, I would change almost nothing.

[lifemean] The Meaning in Life Questionnaire [79]

Please take a moment to think about what makes your life feel important to you during the COVID-19 pandemic. Please respond to the following statements as truthfully and accurately as you can, and also please remember that these are very subjective questions and that there are no right or wrong answers. Please answer according to the scale below:

*Responses were recorded on a scale from 1 (Absolutely untrue) to 7 (Absolutely true)*

[lifemean1] I understand my life’s meaning.

[lifemean4] My life has a clear sense of purpose.

[lifemean5] I have a good sense of what makes my life meaningful.

[lifemean6] I have discovered a satisfying life purpose.

[lifemean9] My life has no clear purpose.

[anxiety] Perceived Stress Scale [81]

The questions in this scale ask you about your feelings and thoughts during COVID-19 pandemic. In each case, you will be asked to indicate how often you felt or thought a certain way. Although some of the questions are similar, there are differences between them and you should treat each one as a separate question. The best approach is to answer each question fairly quickly. That is, don't try to count up the number of times you felt a particular way, but rather indicate the alternative that seems like a reasonable estimate. For each question choose from the following alternatives:

*Responses were recorded on a scale from 1 (Never) to 5 (Very often)*

[anxiety1] During the pandemic, how often have you been upset because of something that happened unexpectedly?

[anxiety2] During the pandemic, how often have you felt that you were unable to control the important things in your life?

[anxiety3] During the pandemic, how often have you felt nervous and "stressed"?

[anxiety4] During the pandemic, how often have you dealt successfully with irritating life hassles?

[anxiety5] During the pandemic, how often have you felt that you were effectively coping with important changes were occurring in your life?

[anxiety6] During the pandemic, how often have you felt confident about your ability to handle your personal problems?

[anxiety7] During the pandemic, how often have you felt that things were going your way?

[anxiety8] During the pandemic, how often have you found that you could not cope with all the things that you had to do?

[anxiety9] During the pandemic, how often have you been able to control irritations in your life?

[anxiety10] During the pandemic, how often have you felt that you were on top of things?

[anxiety11] During the pandemic, how often have you been angered because of things that happened that were outside of your control?

[anxiety12] During the pandemic, how often have you found yourself thinking about things that you have to accomplish?

[anxiety13] During the pandemic, how often have you been able to control the way you spend your time?

[anxiety14] During the pandemic, how often have you felt difficulties were piling up so high that you could not overcome them?
